# A systematic review and meta-analysis of the global prevalence and relationships among *Burkholderia pseudomallei* sequence types isolated from humans, animals, and the environment

**DOI:** 10.14202/vetworld.2024.26-36

**Published:** 2024-01-04

**Authors:** Sa-ngob Laklaeng, Doan Hoang Phu, Jirarat Songsri, Sueptrakool Wisessombat, Wanida Mala, Wilaiwan Senghoi, Preeda Phothaworn, Manit Nuinoon, Tuempong Wongtawan, Wiyada Kwanhian Klangbud

**Affiliations:** 1 College of Graduate Studies, Walailak University, Nakhon Si Thammarat 80160, Thailand; 2 Faculty of Animal Science and Veterinary Medicine, Nong Lam University, Ho Chi Minh City 70000, Vietnam; 3 Center of Excellence in Research for Melioidosis and Microorganisms, Walailak University, Nakhon Si Thammarat 80160, Thailand; 4 Department of Medical Technology, School of Allied Health Sciences, Walailak University, Nakhon Si Thammarat 80160, Thailand; 5 Akkhraratchakumari Veterinary College, Walailak University, Nakhon Si Thammarat 80160, Thailand

**Keywords:** *Burkholderia pseudomallei*, melioidosis, meta-analysis, One Health, sequence type, systematic review

## Abstract

**Background and Aim:**

*Burkholderia pseudomallei*, a highly pathogenic bacterium responsible for melioidosis, exhibits ecological ubiquity and thrives within soil and water reservoirs, posing significant infection risks to humans and animals through direct contact. The aim of this study was to elucidate the genetic diversity and prevalence patterns of *B. pseudomallei* sequence types (STs) across a global spectrum and to understand the relationships between strains isolated from different sources.

**Materials and Methods:**

We performed a systematic review and meta-analysis in this study. Extensive research was carried out across three comprehensive databases, including PubMed, Scopus, and ScienceDirect with data collected from 1924 to 2023.

**Results:**

A total of 40 carefully selected articles contributed 2737 *B. pseudomallei* isolates attributed to 729 distinct STs and were incorporated into the systematic review. Among these, ST46 emerged as the most prominent, featuring in 35% of the articles and demonstrating a dominant prevalence, particularly within Southeast Asia. Moreover, ST51 consistently appeared across human, animal, and environmental studies. Subsequently, we performed a meta-analysis, focusing on nine specific STs: ST46, ST51, ST54, ST70, ST84, ST109, ST289, ST325, and ST376. Surprisingly, no statistically significant differences in their pooled prevalence proportions were observed across these compartments for ST46, ST70, ST289, ST325, and ST376 (all p > 0.69). Conversely, the remaining STs, including ST51, ST54, ST84, and ST109, displayed notable variations in their prevalence among the three domains (all p < 0.04). Notably, the pooled prevalence of ST51 in animals and environmental samples surpassed that found in human isolates (p < 0.01).

**Conclusion:**

To the best of our knowledge, this study is the first systematic review and meta-analysis to investigate the intricate relationships between STs and their sources and contributes significantly to our understanding of *B. pseudomallei* diversity within the One Health framework.

## Introduction

*Burkholderia pseudomallei* is a Gram-negative, oxidase-positive, motile, and facultative intracellular bacterium that has been classified by the United States Centers for Disease Control and Prevention as the causative agent of melioidosis in both humans and select animals, including pigs, horses, and cattle, placing it in the highest-risk category as a tier 1 select agent [1]. It is commonly found in tropical and subtropical environments, such as soil, water, and agricultural fields, and exhibits remarkable resilience to high temperatures, wide pH ranges, and malnutrition [2–4]. Recent studies have reported survival in distilled water for up to 16 years [5]. The discovery of *B. pseudomallei* in Myanmar dates back to 1911, when Whitmore and Krishnaswami first identified it [2]. Globally, certain regions, such as Australia’s Northern Territory and north-eastern Thailand, are considered endemic areas with the highest incidence of melioidosis [6]. In recent years, melioidosis cases have increased in other regions, such as the Middle East, Central and South America, Africa, and the Caribbean [7]. Melioidosis accounts for an estimated 165,000 cases and 89,000 deaths annually [8]. *B. pseudomallei* infects humans and animals through various routes, including inoculation through open wounds, inhalation of droplets during the wet season, particularly after monsoons, and ingesting food contaminated with pathogens. At present, no melioidosis vaccine exists, and *B. pseudomallei* has shown resistance to the host’s innate immune response [2–4]. The previous epidemiological studies have identified several risk factors, such as diabetes, alcoholism, lung disease, and chronic kidney disease, that increase susceptibility and mortality to melioidosis infection [3, 6]. Melioidosis presents diverse clinical manifestations, from localized organ abscesses to disseminated systemic disease, which shares similarities with pneumonia and tuberculosis [6].

Multilocus sequence types (MLST) is a widely adopted methodology for evaluating the epidemiological spread of bacterial pathogens, allowing for the differentiation of various strains [9]. This technique plays a crucial role in elucidating relationships between bacterial strains and monitoring the global dissemination of diverse bacterial species [10]. MLST employ seven specific housekeeping genes (*ace*, *gltB*, *gmhD*, *lepA*, *lipA*, *narK*, and *ndh*) to represent the multi-allele genetics employed for categorizing bacteria in *B. pseudomallei* studies [11]. MLST have been instrumental in identifying the diversity and distribution of *B. pseudomallei* across clinical, animal, and environmental samples, which is essential in epidemiological research. Some specific STs of *B. pseudomallei* have been associated with more severe clinical outcomes and increased virulence in certain regions. For instance, ST46, ST51, and ST84 have garnered attention due to their association with increased mortality in cases of melioidosis, particularly prevalent in parts of Southeast Asia, including Thailand and Australia [12]. While recent systematic reviews and meta-analyses related to *B. pseudomallei* have focused on specific aspects, such as assessing the accuracy of melioidosis diagnosis tests [13], investigating cardiac manifestations [14], or identifying environmental determinants influencing the distribution of *B. pseudomallei* [15], a research gap exists concerning the relationship between STs and diverse source isolation.

Therefore, in this study, we conducted a systematic review and meta-analysis of various *B. pseudomallei* STs identified worldwide. We assessed the prevalence of STs across three compartments: Human clinical isolates, animals, and the environment through a meta-analysis, we compared the frequency of prominent STs obtained from different sample sources under the One Health approach, encompassing humans, animals, and the environment. The findings of the present study contribute to our enhanced understanding of the diversity and dissemination of *B. pseudomallei*, providing valuable insights for further investigations into *B. pseudomallei* surveillance systems aimed at combating melioidosis in human infections.

## Materials and Methods

This systematic review was conducted in accordance with Preferred Reporting Items for Systematic Reviews and Meta-Analyses guidelines [16].

### Study period and location

The literature search was conducted in January 2023 at Walailak University, Nakhon si Thammarat, Thailand.

### Search strategy

A comprehensive literature search (from 1924 to 2023) was conducted using PubMed (https://pubmed.ncbi.nlm.nih.gov/), Scopus (https://www.scopus.com), and ScienceDirect (https://www.sciencedirect.com/) databases. Search terms were developed in consultation with a specialist medical librarian and a professor with expertise in bacteriology. We employed the following search terms using Boolean operators “AND” and “OR”: “MLST” OR “Multilocus sequence typing” OR “sequence typing” OR “STs” AND “*Burkholderia pseudomallei*” OR “*B. pseudomallei*” AND “melioidosis.” In addition, a manual search of the reference lists of selected articles has been carried out to extend the scope of articles eligible for inclusion.

### Inclusion and exclusion criteria of selected studies

To identify relevant articles, the first step in study selection involved searching and screening titles and abstracts. Articles included must describe the prevalence of *B. pseudomallei* and its STs in the abstract and be selected for further evaluation through a full-text review. *B. pseudomallei* isolates detected from samples collected from (1) humans and (2) animals (all animal species) and the environment (water, soil, drainage, and sludge) were reported. Articles that did not contain sufficient information on *B. pseudomallei*, prevalence data, STs, or disaggregated data on the host species were excluded from the study. In addition, review articles, conference abstracts, book chapters, letters, and articles written in languages other than English were excluded from this study. The two authors independently conducted the selection process. Article selection adhered to the Joanna Briggs Institute Critical Appraisal Checklist, specifically designed for studies reporting prevalence data [16]. A third reviewer was involved to resolve any disagreements regarding article inclusion in cases where discrepancies arose in study selection between the two reviewers. No specific restrictions have been applied, except for the exclusion of duplicate articles from databases, review articles, and articles lacking full-text when retrieving data using EndNote X20 (Clarivate Analytics, Philadelphia, PA, U.S.A).

### Data extraction

A structured Microsoft Excel (Microsoft Corporation, Redmond, WA, U.S.A.) spreadsheet was used to systematically collect data from the selected studies. To mitigate selection bias, two authors independently participated in data extraction and cross-checked the extracted data for consistency. In the event of discrepancies during data collection, the third reviewer was responsible for making the final decision. We extracted study details (authors), country of research, sample size, sample sources (clinical, environment, and/or animals), year of enrollment, specific methods used for collecting clinical isolates (e.g., blood, sputum, pus, and urine), and sequencing types (total number of each ST reported) from the articles.

### Statistical analysis

As this study aimed to investigate ST relationships within the One Health framework, only studies reporting ST data across the compartments of humans, animals, and the environment, with a minimum of two articles, were included for meta-analysis. Prevalence data for STs were calculated as percentages representing the fraction of the total STs reported in each study for each ST (summing up to hundred percentage). To identify within-study variance (VYi) and estimate between-studies variance (τ2), we used a generalized linear mixed-effect model with a random-effect model [17, 18]. Heterogeneity among selected studies was assessed using the inverse variance index (I^2^), with values of 25%, 50%, and 75% indicating low, medium, and high heterogeneity, respectively [19]. The significance was determined at p = 0.05. We conducted subgroup analyses to investigate the prevalence of ST among the three sources. The results of meta-analysis and subgroup analysis were visually represented using forest plots. The publication bias was assessed using contour-enhanced funnel plots and Egger’s regression test. Meta-analysis, subgroup analyses, and publication bias testing were performed using the “meta” and “metafor” packages in R software (R Foundation for Statistical Computing, Vienna, Austria), whereas publication bias assessment was performed using the “tidyverse” package (https://www.tidyverse.org/packages/).

## Results

### Article selection process

**Figure-1:**
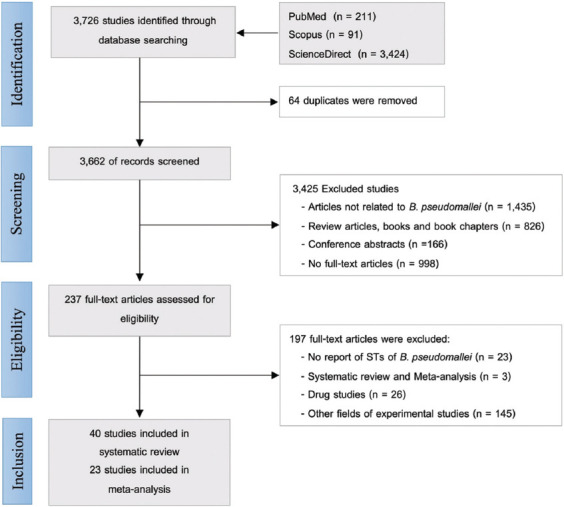
Preferred Reporting Items for Systematic Reviews and Meta-analyses flow diagram of studies’ screening and selection.

A total of 3726 studies were identified in the Science Direct, PubMed, and Scopus databases. Among these, 64 duplicate studies were removed, leaving 3662 studies for title and abstract screening. We excluded 1435 articles, 826 books, book chapters, review articles, and 998 non-full-text articles during this phase. Subsequently, 237 full-text papers were reviewed for eligibility. Within this phase, we excluded 197 articles, including 23 that did not present *B. pseudomallei* ST data, 171 that conducted clinical trials and pharmacological research without ST-related findings, and three systematic reviews and meta-analysis studies. In the systematic review, 40 articles remained for qualitative synthesis (Supplementary-S1). Among these, 23 studies representing *B. pseudomallei* STs across human, animal, and environmental compartments were selected for quantitative synthesis in the meta-analysis (Figure-1).

### Characteristics of included studies

**Figure-2:**
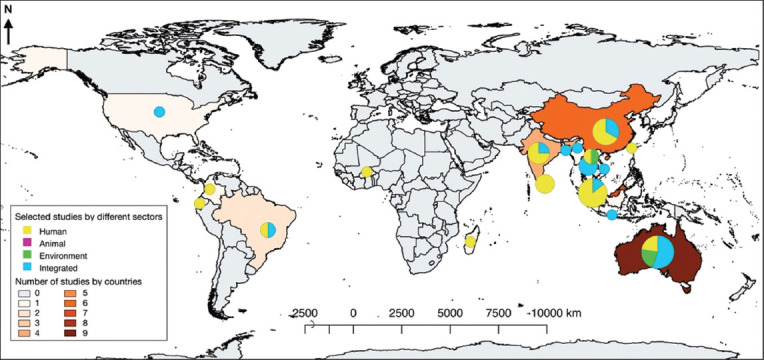
The global prevalence of multi-locus sequence types studies conducted in humans, animals, and the environment. The global studies on *Burkholderia pseudomallei* were visualized using the QGIS software version 2.18.15 (QGIS Association; https://www.qgis.org); the layers of the figure were downloaded from DIVA-GIS (https://www.diva-gis.org/).

Among the 40 studies selected for the systematic review, 19 (47.5%) focused on humans, 4 (10%) focused on environmental aspects (10.0%), 17 (42.5%) were classified as integrated studies, 12 (30.0%) focused on humans and the environment, 1 (2.5%) focused on animals and the environment, and 4 (10.0%) were conducted across all three compartments. The majority of selected studies have been conducted in the last decade, mostly from 2011 to 2022 (97.5%). Regarding the study area, the primarily selected studies were retrieved in databases from 12 countries in the endemic area of Asia (28/40, 70.0%), in which 14 studies (35.0%) were found in Southeast Asia [8, 12, 20–31], and seven studies (17.5%) were found in South Asia [19, 32–37] and East Asia [38–44]. Nine studies were conducted in Australia (22.5%) [45–53], and three studies were performed in North, South, and Central America (7.5%) [54–56] (Figure-2). Regarding *B. pseudomallei* obtained from animal studies conducted in Australia and Southeast Asia involving species such as horses, pigs, cats, iguanas, parrots, and goats. Samples collected in human and animal studies included blood, pus, sputum, sanies, abscesses, and body fluids, whereas soil and water were collected in environmental studies. Table-1 presents the detailed characteristics of the included studies.

**Table-1:** Characteristics of selected studies.

Characterization of included study	Human study n (%)	Environment study n (%)	Animal study n (%)	Integrated study			Total n (%)

				Human and environment n (%)	Environment and animal n (%)	Human, environment, and animal n (%)	
Number of studies							
	19 (47.5)	4 (10.0)	0 (0)	12 (30.0)	1 (2.5)	4 (10.0)	40 (100.0)
Year of publication							
2001–2010	-	-	-	-	-	1 (2.5)	1 (2.5)
2011–2020	16 (40.0)	3 (7.5)	-	9 (22.5)	1 (2.5)	3 (7.5)	32 (80.0)
2021–2022	3 (7.5)	1 (2.5)	-	3 (7.5)	-	-	7 (17.5)
Study area							
North, South and Central America	2 (5.0)	-	-	1 (2.5)	-	-	3 (7.5)
Australia	-	2 (5.0)	-	4 (10.0)	-	3 (7.5)	9 (22.5)
Southeast Asia	8 (20.0)	1 (2.5)	-	3 (7.5)	1 (2.5)	1 (2.5)	14 (35.0)
East Asia	3 (7.5)	1 (2.5)	-	3 (7.5)	-	-	7 (17.5)
South Asia	6 (15.0)	-	-	1 (2.5)	-	-	7 (17.5)
Sample type							
Blood, pus, sputum, sanies, abscess, body fluids	19 (47.5)	-	-	12 (30.0)	-	4 (10.0)	35 (87.5)
Water, soil	-	2 (5.0)	-	-	-	-	2 (5.0)
Water	-	1 (2.5)	-	-	-	-	1 (2.5)
Soil	-	1 (2.5)	-	-	-	-	1 (2.5)
Organ abscess, water, soil	-	-	-	-	1 (2.5)	-	1 (2.5)
Diversity of ST							
<10 STs	7 (17.5)	2 (5.0)	-	4 (10.0)	1 (2.5)	1 (2.5)	15 (37.5)
10–20 STs	4 (10.0)	2 (5.0)	-	4 (10.0)	-	1 (2.5)	11 (27.5)
21–30 STs	4 (10.0)	-	-	1 (2.5)	-	-	5 (12.5)
31–40 STs	2 (5.0)	-	-	1 (2.5)	-	-	3 (7.5)
>40 STs	2 (5.0)	1 (2.5)	-	1 (2.5)	-	2 (5.0)	6 (15.0)

ST=Sequence type

### The diversity of STs of *B. pseudomallei* among selected studies

All 40 studies included a total of 2737 isolates from three compartments: humans, animals, and environmental resources. The median number of *B. pseudomallei* isolates was 18 (interquartile range 6−60, min−max 1−736 isolates). A total of 729 distinct STs were documented in this review. Notably, ST46 was the most prevalent, featuring in 14 articles, with a predominant presence in Southeast Asia. ST51 and ST54 each appeared in nine articles, while ST70 and ST376 each appeared in seven articles. Several other STs, including ST10, ST84, ST109, ST289, ST325, and ST376, have been reported in six articles. The remaining STs were rarely reported in the selected studies. With regard to the diversity of recorded STs, 15 studies (37.5%) found fewer than 10 STs in their investigations. Meanwhile, 11 studies (27.5%) found 10−20 distinct STs in their findings. Five studies (12.5%) identified 21−30 STs, whereas three studies detected 31−40 STs. In addition, six studies provided comprehensive reports with more than 40 distinct STs documented within their research (Table-1).

### The pooled prevalence of *B. pseudomallei* STs in meta-analysis

**Figure-3:**
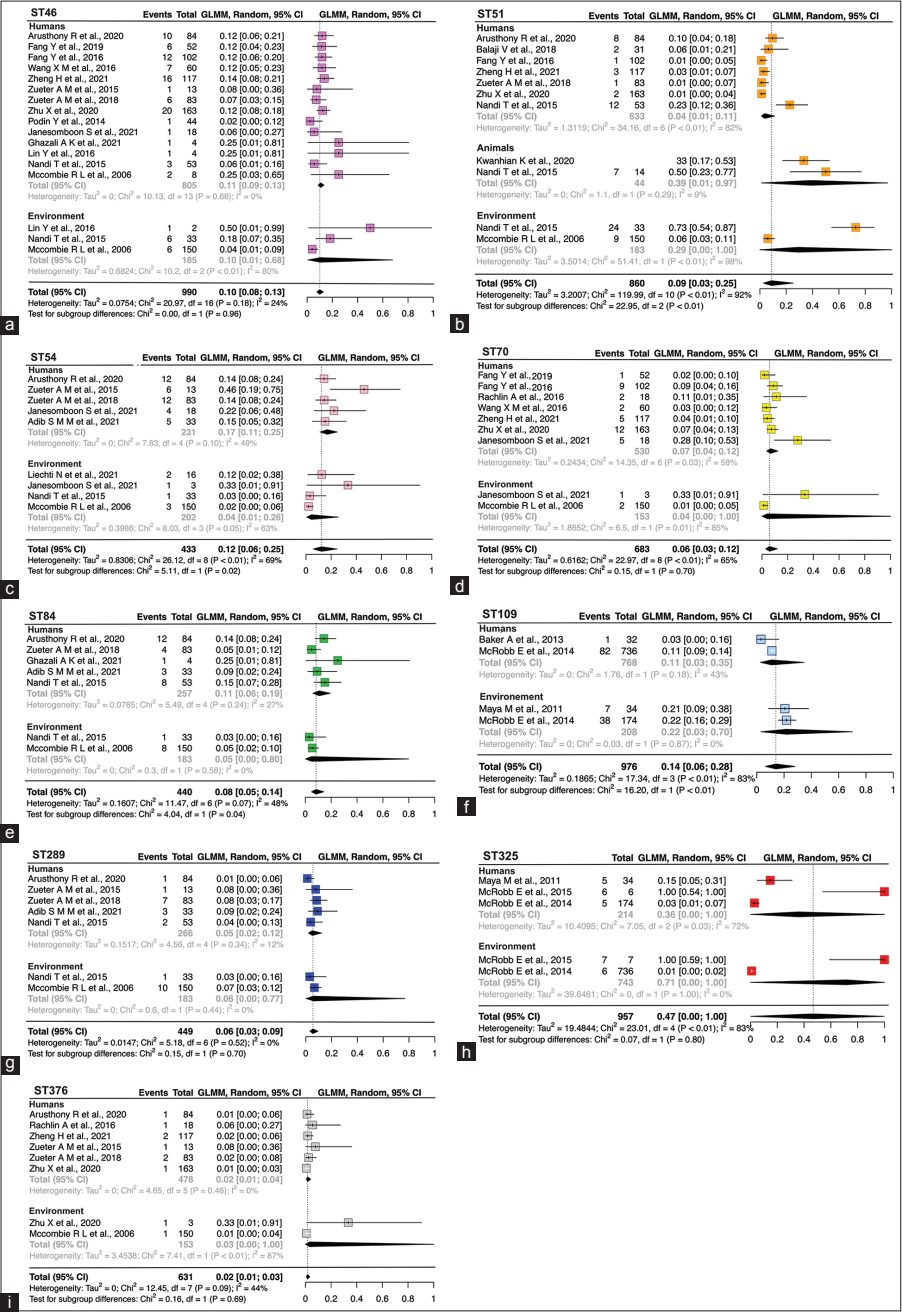
(a-i) Forest plot of the pooled prevalence of *Burkholderia pseudomallei* nine sequence types (ST46, ST51, ST54, ST70, ST84, ST109, ST289, ST325, and ST376).

In the meta-analysis, only STs identified in more than two articles across humans, animals, and the environment compartments were eligible for inclusion. A total of 23 articles, including ST46, ST51, ST54, ST70, ST84, ST109, ST289, ST325, and ST376, were selected for this phase. Notably, ST51 was the only ST present across all three compartments, whereas the remaining eight STs were exclusively detected in humans and environmental studied. Figure-3 shows visual representations of forest plots for the selected STs.

Seventeen studies have reported findings on ST46, with 14 studies focusing on humans and 3 on environmental samples. The crude prevalence (standard error [SE] ± 3.0) was 16.0%, whereas the adjusted prevalence after the meta-analysis was 10.0% (95% confidence interval [CI] 1.0%−68.0%). Subgroup analysis showed no significant difference between humans and the environment (p = 0.96) (Table-2 and Figure-3a).

**Table-2:** The pooled prevalence of subgroup mete-analysis of nine common STs isolated from humans, animals, and the environment.

STs	Compartments			Total

	Human	Environment	Animal	
ST46				
Pooled prevalence (%)	11.0	10.0	_	10.0
95% CI	(9.0–13.0)	(1.0–68.0)	_	(8.0–13.0)
p-value	0.68	<0.01	_	0.96
Number of studies	14 [12, 22–23, 25–27, 29, 38–43, 53]	3 [39, 41, 53]	_	17
ST51				
Pooled prevalence (%)	4.0	29.0	39.0	9.0
95% CI	(1.0–11.0)	(0.0–100.0)	(1.0–97.0)	(3.0–25.0)
p-value	<0.01	<0.01	0.29	<0.01
Number of studies	7 [12, 19, 23, 39, 40, 42, 53]	2 [30, 53]	2 [29, 53]	11
ST54				
Pooled prevalence (%)	17.0	4.0	_	12.0
95% CI	(11.0–25.0)	(1.0-26.0)	_	(6.0-25.0)
p-value	0.10	0.05	_	0.02
Number of studies	5 [12, 22–23, 26, 28]	4 [20, 26, 29, 53]	_	9
ST70				
Pooled prevalence (%)	7.0	4.0	_	6.0
95% CI	(4.0–12.0)	(0.0–100.0)	_	(3.0–12.0)
p-value	0.03	0.01	_	0.70
Number of studies	7 [21, 26, 38–40, 41–42]	2 [26, 29]	_	9
ST84				
Pooled prevalence (%)	11.0	5.0	_	8.0
95% CI	(6.0–19.0)	(0.0–80.0)	_	(5.0–14.0)
p-value	0.24	0.58	_	0.04
Number of studies	5 [12, 23, 27–28, 53]	2 [29, 53]	_	7
ST109				
Pooled prevalence (%)	11.0	22.0	_	14.0
95% CI	(3.0–35.0)	(3.0–70.0)	_	(6.0–28.0)
p-value	0.18	0.87	_	<0.01
Number of studies	2 [46, 51]	2 [48, 51]	_	4
ST289				
Pooled prevalence (%)	5.0	6.0	_	6.0
95% CI	(2.0–12.0)	(0.0–77.0)	_	(3.0–9.0)
p-value	0.34	0.44	_	0.70
Number of studies	5 [12, 22, 23, 28, 53]	2 [29, 53]	_	7
ST325				
Pooled prevalence (%)	36.0	71.0	_	47.0
95% CI	(0.0–100.0)	(0.0–100.0)	_	(0.0–100.0)
p-value	0.03	1.00	_	0.80
Number of studies	3 [48, 49, 51]	2 [49, 51]	_	5
ST376				
Pooled prevalence (%)	2.0	3.0	_	2.0
95% CI	(1.0–4.0)	(0.0–100.0)	_	(1.0–3.0)
p-value	0.46	<0.01	_	0.69
Number of studies	6 [12, 21–23, 40, 43]	2 [29, 40]	_	8

STs=Sequence types, CI=Confidence interval

Eleven studies presented data on ST51, including seven on humans, two on animals, and two on the environment. The crude prevalence was 18.8 (SE ± 7.2), whereas the pooled prevalence was 9.0% (95% CI 3.0%−25.0%). Notably, there was a significant difference in pooled prevalence among humans, animals, and the environment. The highest prevalence (39.0%) was observed in animals, followed by the environment (29.0%), with a notably lower pooled prevalence of 4.0% in humans (p < 0.01) (Figure-3b).

Nine studies included ST54 data, with five focusing on humans and four on the environment. The crude prevalence (SE±4.7) was 18.1 and the pooled prevalence was 12.0 (95% CI 6%−25.0%). A significant difference was observed in the pooled prevalence of ST54 between humans (17%) and the environment (4.0%) (p = 0.02) (Figure-3c).

Similarly, nine studies have reported data regarding ST70 (Figure-3d), with seven involving humans and two related to the environment. The crude prevalence (SE ± 3.8) was 11.0, whereas the pooled prevalence was slightly higher 6.0 (95% CI 3.0%−12.0%). Importantly, there was no significant difference in the pooled prevalence of ST70 between humans and environment.

Seven studies contributed to the prevalence data for ST84 in our review, with five focusing on humans and two focusing on the environment (Figure-3e). The crude prevalence was estimated to be 10.9 (SE ± 2.9), whereas the pooled prevalence was estimated to be 8.0% (95% CI 5%−14%). There was a significant difference in the pooled prevalence among humans (11.0%) and the environment (5.0%) (p = 0.04).

Data on ST109 has been reported in four studies, with two studies focusing on each compartment of humans and the environment. The crude prevalence was estimated to be 14.2% (SE ± 4.4), whereas the adjusted prevalence was also similar, at 14.0% (95% CI 6%−28%). Subgroup analyses revealed a significant difference in the pooled prevalence of ST109 between humans (11.0%) and the environment (22.0%) (p < 0.01) (Table-2 and Figure-3f).

Our review included seven studies reporting the prevalence of ST289, with five focusing on humans and two focusing on the environment (Figure-3g). The crude prevalence was found to be 3.0 (95% CI 3.0%−9.0%) (SE ± 1.1), while the pooled prevalence after meta-analysis was slightly higher 6.0%. However, no significant difference was observed in the prevalence of ST289 between humans and environment (p = 0.70).

ST325 has been reported in four studies, with two studies on humans and two on the environment. The crude prevalence of ST325 across these four studies was calculated as 43.7 (SE ± 23.11), with a pooled prevalence of 47.0% (95% CI 0.0%−100.0%) after meta-analysis. Notably, no significant difference was observed in pool prevalence of ST325 between humans and the environment (p = 0.80) (Figure-3h).

The last ST in our meta-analysis, ST376, has been reported in five studies involving humans and two studies related to the environment. The crude prevalence was calculated to be 6.7 (SE ± 3.9), while the pooled prevalence was slightly lower at 2.0% (95% CI 1.0%−3.0%). No significant difference was found in the pooled prevalence of ST376 between humans and the environment (p = 0.69) (Figure-3i).

### Publication bias

**Figure-4:**
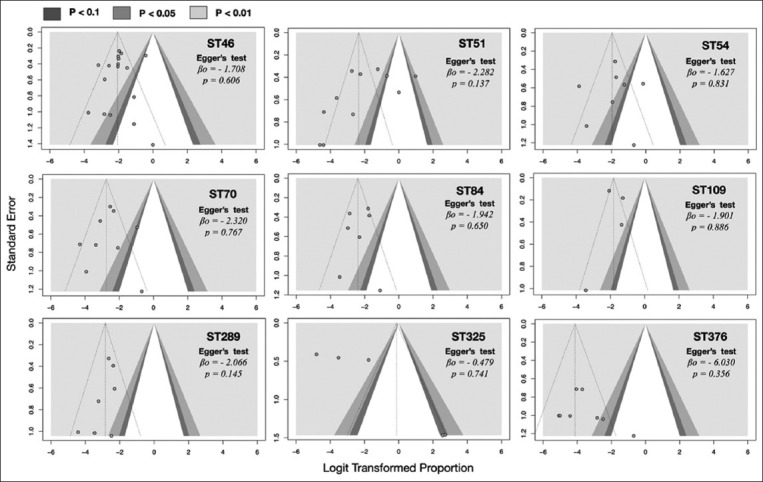
Funnel plot of *Burkholderia pseudomallei* nine sequence types (ST46, ST51, ST54, ST70, ST84, ST109, ST289, ST325, and ST376).

Publication bias assessment indicated that almost all nine funnel plots showed asymmetry, with scatters unevenly distributed in the graphs (Figure-4). Egger’s test findings, aimed at quantitatively determining publication bias by testing funnel plot asymmetry, indicated that the intercept (β_0_) differed from zero for all selected STs. Only ST325 had an Egger’s intercept test equal to zero value (β_0_ = −0.479); however, it did not reach statistical significance (p = 0.741), confirming the presence of funnel plot asymmetry, which suggests publication bias.

## Discussion

One Health is an integrated approach that focuses on health issues, including human, animal, plant, environment, and ecosystem [57]. The term “One Health” gained prominence during the severe acute respiratory syndrome outbreak in 2003–2004 and further significance during the global COVID-19 pandemic in 2019 [58]. *B. pseudomallei*, the causative agent of melioidosis, resides in environmental niches, specifically in water [59]. The previous studies have consistently demonstrated that direct exposure to these environmental reservoirs significantly increases the risk of melioidosis in humans and animals. The risk of melioidosis infection is heightened in individuals engaged in agricultural work who frequently come into contact with soil and water sources [3].

The primary findings from the qualitative and quantitative syntheses in our review are as follows: (1) a wide diversity of MLST types was identified across studies encompassing human, animal, and environmental sources worldwide; (2) despite our focus on a subset of STs of only nine STs in the meta-analyses, we observed no significant disparity in the distribution of *B. pseudomallei* STs between humans and the environment among STs of *B. pseudomallei*, specifically ST46, ST70, ST289, ST325, and ST376; and (3) while ST51 was detected across all three compartments, our analysis revealed a significantly higher pooled prevalence of ST51 in animals and the environment compared to its prevalence in humans.

Our findings revealed a remarkable diversity of STs across three distinct compartments of humans, animals, and the environment, encompassing 729 distinct STs of *B. pseudomallei* worldwide. It has been reported that there are different levels of diversity in specific regions. A previous study in Australia reported a diversity ratio of 0.65 STs per isolate [2], whereas two studies conducted in Malaysia reported diversity ratios of 0.35 and 0.38 STs per isolate, respectively [12, 60]. On the other hand, a study in Thailand documented a notably lower diversity among 630 *B. pseudomallei* strains, characterizing only 7 STs [61]. At present, little is known about the evidence linking specific STs to mortality, virulence, or clinical presentation [11]. Ghazali *et al*. [27] compared *B. pseudomallei* low-virulence gene strains with high-virulence gene strains and suggested that gene loss in low-virulence gene strains may be associated with pathogen survival and adaptation within the host cell. These results highlight the significance of factors (i.e., fitness costs) in the adaptation and evolution mechanisms of bacteria. Given that STs of bacteria are typically associated with genotype and phenotype characteristics, it is recommended that further investigations explore the potential relationships between STs and factors such as mortality, virulence, or clinical presentation in *B. pseudomallei*.

Although our analyses focused on a limited subset of nine STs, to some degree, our findings revealed a marginal discrepancy in the distribution of *B. pseudomallei* STs between humans and the environment. These results suggest that STs of *B. pseudomallei* tend to be equally prevalent across compartments, including humans and the environment. These results highlight the possibility of transmitting infections to humans through environmental sources. A recent study in Thailand reported a high risk of *B. pseudomallei* infection in workers in rice farming in Thailand who often spend extended periods in rice fields without adequate protective equipment [62]. *B. pseudomallei* is a pathogenic bacterium that can infect individuals through skin abrasions or inhalation of contaminated water droplets, particularly during the monsoon season [63]. The previous studies have also indicated that stormwater, which accumulates various substances such as particles, minerals, contaminants, and bacteria from the soil, might serve as a more accurate indicator of catchment areas and distribution of *B. pseudomallei* [64, 65]. Experiments exploring the environmental distribution of *B. pseudomallei* suggest that surface runoff and stormwater analysis could be more effective than random soil sampling in identifying melioidosis endemic regions [19, 65].

ST51 consistently appeared in all three compartments. This ST is notably prevalent in Australia and Bangladesh and frequently encountered in Southeast Asia [12]. Our analysis revealed a significantly higher pooled prevalence of ST51 in animals and the environment than in humans. These findings suggest that specific STs, such as ST51, are prevalent among humans, animals, and the environment, potentially indicating host specificity for animals. Given the challenges associated with the detection of *B. pseudomallei* in animal populations due to limitations in diagnostic methods and asymptomatic carriage in animals, further investigation of the dissemination of melioidosis in animal populations is warranted in the future.

We acknowledge the limitations regarding the number of studies available for inclusion across all compartments, particularly the restricted representation of studies in animals, which prevents a comprehensive assessment of *B. pseudomallei* prevalence. In a previous retrospective study, the susceptibility to melioidosis has been demonstrated in a wide range of animal species, including pigs, sheep, goats, and cattle. This poses a significant risk for humans, especially for those working with animals, such as herders, veterinarians, slaughterhouse workers, and consumers of animal products [66]. Therefore, further investigations involving all three compartments, especially in animals, should be carried out using a more comprehensive approach.

In addition, it should be noted that our analysis included some studies that focused on specific cases of human melioidosis, sometimes involving only a limited number of cases. Our calculations are based on the proportion of STs relative to the total sample size in these smaller studies. If random sampling methods were used in the studies, a more accurate estimation of prevalence could be achieved. This discrepancy is responsible for the observed disparity between the pooled and crude prevalence and highlights the influence of sample weights from selected studies on the pooled effect size. In addition, the substantial heterogeneity observed in our review may indicate publication bias, potentially stemming from the impact of these smaller studies included in our analysis.

## Conclusion

This systematic review and meta-analysis from the perspective of One Health sheds light on the diversity and relationships among *B. pseudomallei* strains globally. Our study significantly enhances our understanding of STs within this pathogenic bacterial species. In addition, this study highlights the enduring importance of MLST data for ongoing outbreak surveillance and provides a comprehensive understanding of the epidemiological implications associated with *B. pseudomallei* distribution. Future studies using the One Health approach, including humans, animals, and the environment, should explore molecular genotyping molecular genetics to deepen our understanding of *B. pseudomallei*.

## Data Availability

Supplementary data can be available from the corresponding author on a reasonable request.

## Authors’ Contributions

SL and WKK: Designed the study. SL, DHP and WKK: Performed papers searching, selection and extraction. SL, DHP, JS, SW, WM, WS, PP, MN, TW, and WKK: Performed data analysis and results interpretation. SL, DHP, and WKK: Drafted the manuscript. All authors have read, reviewed, and approved the final manuscript.
